# Characterization and phylogenetic analysis of the complete chloroplast genome of *Cyanthillium cinereum* (L.) H. Rob

**DOI:** 10.1080/23802359.2022.2080602

**Published:** 2022-06-14

**Authors:** Tin-Yan Siu, Kwan-Ho Wong, Bobby Lim-Ho Kong, Hoi-Yan Wu, Grace Wing-Chiu But, Pang-Chui Shaw, David Tai-Wai Lau

**Affiliations:** a Shiu-Ying Hu Herbarium, School of Life Sciences, The Chinese University of Hong Kong, Hong Kong, Hong Kong; b Li Dak Sum Yip Yio Chin R & D Centre for Chinese Medicine, The Chinese University of Hong Kong, Hong Kong, Hong Kong; c School of Life Sciences, The Chinese University of Hong Kong, Hong Kong, Hong Kong; d State Key Laboratory of Research on Bioactivities and Clinical Applications of Medicinal Plants (CUHK) and Institute of Chinese Medicine, The Chinese University of Hong Kong, Hong Kong, Hong Kong

**Keywords:** *Cyanthillium*, *Vernonia*, Vernonioideae, Compositae, phylogenetic analysis

## Abstract

*Cyanthillium cinereum* is a member of the tribe Vernonieae from the family Compositae. The tribe was traditionally placed in the subfamily Cichorioideae, but is recently proposed to be placed in its own subfamily Vernonioideae. The complete chloroplast genome (cp genome) of the genus *Cyanthillium* is sequenced for the first time. The cp genome of *C. cinereum* is 152,750 bp in length. It contained a large single copy (LSC) region (83,871 bp), and small single copy (SSC) region (18,487 bp), and two inverted repeats (IRs, 25,196 bp). Phylogenetic analysis of 20 species was conducted. *C. cinereum* and *Gymnanthemum amygdalinum* which are members of tribe Vernonieae nested outside of the monophyletic clade formed by members of subfamily Cichorioideae. The findings would be useful to understand the phylogeny of the genus *Cyanthillium* and the subfamily Vernonioideae.

## Content

*Cyanthillium cinereum* (L.) H. Rob. (Robinson 1990), formerly *Vernonia cinerea* (L.) Less. (Lessing 1829) and *Conyza cinerea* L. (Linnaeus 1753), is a herb with anti-inflammatory property (Youn et al. 2012), cancer treatment property (Pratheeshkumar and Kuttan 2011; Ariya and Joseph 2020; Amuthan et al. 2021), and is applied in smoking cessation (Puttarak and Bunditanukul 2018).

The species is from the Sunflower Family (Compositae). The species is member of the tribe Vernonieae, also known as the “Evil Tribe” for its taxonomic complexity (Keeley et al. 2007). Phylogenetic analysis supported that the tribe should be separated from the subfamily Cichorioideae to its own subfamily Vernonioideae (Mandel et al. 2019).

The species is characterized by having pale pink tubular florets, and ovate leaves with undulate margin (Strother and Flora of North America Editorial Committee 2006). The specimen of *Cyanthillium cinereum* was cultivated and collected in the campus of the Chinese University of Hong Kong (22.413340°N, 114.209630°E), no special permission is required. The voucher specimen with collector number T. Y. Siu 667 was deposited in the Shiu-Ying Hu Herbarium, School of Life Sciences, the Chinese University of Hong Kong (https://syhuherbarium.sls.cuhk.edu.hk/, David Tai Wai Lau, syhuherbarium.sls@cuhk.edu.hk).

Total genomic DNA of *Cyanthillium cinereum* was extracted from 401 mg of fresh leaves using DNeasy Plant Pro Kit (Qiagen Co., Hilden, Germany) according to the manufacturer’s protocol. Extracted DNA was quantified using NanoDrop Lite (Thermo Fisher Scientific, Massachusetts, USA). The DNA quality was checked by visualization of the DNA by 1% agarose gel electrophoresis. Illumina 150 bp paired-end (PE) library was constructed and sequenced on the NovaSeq 6000 platform (Illumina Inc., San Diego, CA, USA) by Novogene Bioinformatics Technology Co., Ltd. (https://en.novogene.com/, Beijing, China). Poor-quality reads (Phred score < 33) were trimmed using CLC Assembly Cell package v5.1.1 (CLC Inc., Denmark).

The reads were assembled into contigs using the CLC de novo assembler in CLC Assembly Cell package and SOAPdenovo v3.23 with default parameters. Gaps were filled by the Gapcloser module in SOAP package. The contigs were then aligned to the reference genome *Saussurea chabyoungsanica* (NC_036677.1), and assembled into a complete chloroplast genome. Genome annotation was performed on the GeSeq platform by using complete cp genomes of *Saussurea chabyoungsanica* (NC_036677.1) and *Saussurea japonica* (NC_044738.1) as references. A few adjustments for protein-coding genes and start and stop codons were performed manually. The annotated genome was deposited in GenBank with the accession number OK040129.

The cp genome of *Cyanthillium cinereum* was 152,750 bp in length, containing a large single copy (LSC) region (83,871 bp), small single copy (SSC) region (18,487 bp), and two inverted repeats (IRs, 25,196 bp). The GC content was 37.71%. The cp genome contained 111 unique genes, including 80 protein-coding genes, 27 tRNA genes, and 4 rRNA genes.

**Figure 1.**
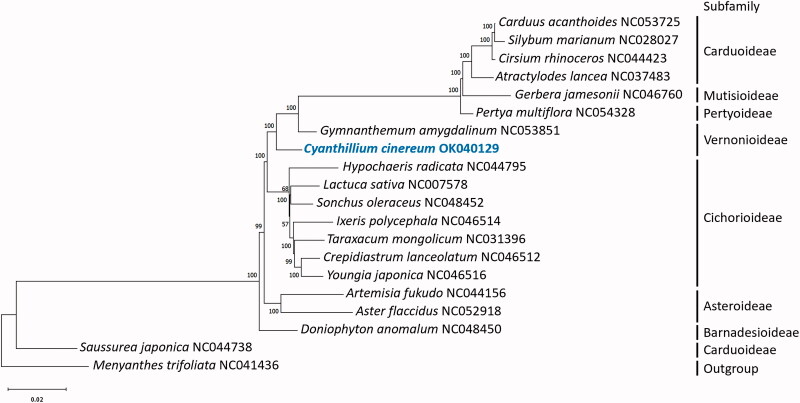
Phylogenetic tree reconstructed by maximum-likelihood (ML) analysis of 20 species.

To investigate the taxonomic position of *Cyanthillium cinereum,* the cp genome was aligned with 18 sequences within Compositae, with *Menyanthes trifoliata* from the closely related family Menyanthaceae as an outgroup. The complete cp genomes were aligned using MAFFT 7.48 (Katoh et al., 2019). A maximum likelihood (ML) tree was constructed using MEGA X (Kumar et al., 2018) based on the best fit model GTR + G and 1000 bootstrap replicates. The phylogenetic tree was labeled with subfamily according to the Global Compositae Database (Compositae Working Group (CWG) 2021) (Figure 1). *Cyanthillium cinereum* and *Gymnanthemum amygdalinum* which are members of tribe Vernonieae nested outside of the monophyletic clade formed by other members of subfamily Cichorioideae, which would support the separation of the tribe from the subfamily Cichorioideae. However, the two species from the tribe Vernonieae, now subfamily Vernonioideae, did not form a monophyletic clade. Further study is required to resolve the intergeneric relationship within the subfamily Vernonioideae and the taxonomic placement of the subfamily Vernonioideae.

## Ethical approval

This research was conducted in accordance with the Legislation of Hong Kong Special Administrative Region. The sample collections did not cause any environmental problem.

## Author contributions

Conceptualization: T.Y.S., D.T.W.L., P.C.S. Methodology: T.Y.S., K.H.W, B.L.H.K, H.Y.W., G.W.C.B. Data analysis: T.Y.S., B.L.H.K, K.H.W. Writing—original draft: T.Y.S. Writing—review and editing: K.H.W, B.L.H.K, H.Y.W., G.W.C.B., D.T.W.L., P.C.S. Supervision: D.T.W.L., P.C.S.

## Data Availability

The data that support the findings of this study are openly available in GenBank (https://www.ncbi.nlm.nih.gov) with the accession number OK040129 (https://www.ncbi.nlm.nih.gov/nuccore/OK040129). The associated BioProject, SRA, and Bio-Sample numbers are PRJNA796482, SRR17635303, and SAMN24865052 respectively.
